# Targeted A-to-G base editing in the organellar genomes of Arabidopsis with monomeric programmable deaminases

**DOI:** 10.1093/plphys/kiad678

**Published:** 2023-12-21

**Authors:** Chang Zhou, Miki Okuno, Issei Nakazato, Nobuhiro Tsutsumi, Shin-ichi Arimura

**Affiliations:** Laboratory of Plant Molecular Genetics, Graduate School of Agricultural and Life Sciences, The University of Tokyo, Tokyo 113-8657, Japan; Division of Microbiology, Department of Infectious Medicine, Kurume University School of Medicine, Fukuoka 830-0011, Japan; Laboratory of Plant Molecular Genetics, Graduate School of Agricultural and Life Sciences, The University of Tokyo, Tokyo 113-8657, Japan; Research Fellow of Japan Society for the Promotion of Science, 5-3-1 Kojimachi, Chiyoda-ku, Tokyo 102-0083, Japan; Laboratory of Plant Molecular Genetics, Graduate School of Agricultural and Life Sciences, The University of Tokyo, Tokyo 113-8657, Japan; Laboratory of Plant Molecular Genetics, Graduate School of Agricultural and Life Sciences, The University of Tokyo, Tokyo 113-8657, Japan

## Abstract

Plastids and mitochondria are 2 intracellular organelles containing DNA-encoding partial but essential components for their roles, photosynthesis, and respiration. Precise base editing in both plastid and mitochondrial genomes would benefit their gene functional analysis and crop breeding. Targeted base editing in organellar genomes relies on a protein-based genome-editing system that uses the TALE-DNA recognition motif with deaminases. This is because the efficient delivery of guide RNA for clustered regularly interspaced short palindromic repeats (CRISPR)/Cas9 systems into organelles is currently impossible. Since TALE-based base editors used in organellar genomes are usually dimeric types, in this study, we used targeted A-to-G base editing in Arabidopsis (*Arabidopsis thaliana*) plastid and mitochondrial genomes with monomeric TALE-based deaminase for easier assembling of vectors. As a result, inheritable targeted A-to-G base editing of adenosine triphosphatase subunit 6-2 (*atp6-2*) in plant mitochondrial genomes and of 16S ribosomal RNA (*16S rRNA*) in plastid genomes of Arabidopsis was successfully induced by monomeric TALE-based adenine deaminase (AD) without off-target mutations. The monomeric TALE-based adenine deaminases also demonstrated a preference for editing the 8th T on the same strand from the recognition end. Phenotypic analysis showed that A-to-G conversion at 1139A of plastid *16S rRNA* conferred substantial spectinomycin resistance in Arabidopsis, but not the other 2 potential-resistant mutations at 1131T and 1137T, predicted from the previous bacterial data. Our study demonstrated the feasibility of monomeric TALE-based ADs in plant organelles and their potential contribution to the functional analyses of plant organelles with easier assembling.

## Teaser

A monomeric programmable base editor achieves targeted A-to-G base editing in both the mitochondrial genome and the chloroplast genome in Arabidopsis.

## Introduction

In addition to the cell nucleus, there is also a portion of essential genetic information located in the organelles of cytoplasm in plants, namely chloroplasts and mitochondria. The targeted editing of organellar DNA is important for uncovering genuine functions of organellar-encoded genes. The clustered regularly interspaced short palindromic repeats (CRISPR)/Cas system has been widely used by scientists for genome editing from basic science to applications such as biomedicine and agriculture since its introduction (Lander 2016). However, it has yet to have an established application in organellar genome editing since it is still difficult to deliver guide RNA into organelles (Gammage et al. 2018; Schmiderer et al. 2022). On the other hand, the protein-only genome-editing enzymes, transcription activator-like effector nucleases (TALENs), are successfully used for editing mammalian or plant mitochondrial genomes by attaching the organelle targeting signals to the N-terminus of the enzymes (Bacman et al. 2013; Kazama et al. 2019). The cytoplasmic male sterility (CMS)–associated genes (ORF 79 [*orf79*] and *orf125*) of CMS varieties of rice (*Oryza sativa* L.) and rapeseed (*Brassica napus* L.), respectively, were successfully knocked out by TALENs with mitochondria localization signals (mitoTALENs; Kazama et al. 2019). Such a nuclease-based knock-out strategy in rice and rapeseed led to large deletions (100bp to 5 kb) and ectopic recombination, which altered the genomic structure and then might cause other potential problems. Compared with knocking out by nucleases, targeted base editing, which mainly involves cytosine base editing (CBE; C:G pairs to T:A pairs, C-to-T) and adenine base editing (ABE; A:T pairs to G:C pairs, A-to-G), allows mild and precise genome editing without double-stranded DNA cleavage causing insertions, deletions, and recombinations. CRISPR/Cas9-based CBEs and ABEs were developed in 2016 (Komor et al. 2016; Nishida et al. 2016) and 2017 (Gaudelli et al. 2017). In 2020, CRISPR-free DdCBE, composed of TALE array proteins, a split DddA half, which is an interbacterial toxin that catalyzes the deamination of cytidines, and a uracil glycosylase inhibitor (UGI), was developed and applied to targeted C-to-T base editing in human mitochondrial DNA (Mok et al. 2020). Based on this TALE-split DddA half-UGI motif, CRISPR-free DdCBEs were then applied to the chloroplast (Kang et al. 2021; Li et al. 2021; Nakazato et al. 2021, 2023) and mitochondrial genomes (Kang et al. 2021; Nakazato et al. 2022) in plants, and an efficient catalysis of C-to-T substitutions in the organelles was reported. In 2022, A-to-G base editing was also reported in mitochondrial DNA from human cells (Cho et al. 2022) and in plant chloroplast DNA (Mok et al. 2022). Plant mitochondrial genomes are known to be AT-rich, and therefore, A-to-G base editing would be more useful, but to our knowledge, targeted A-to-G base editing has yet to be reported in plant mitochondrial genomes. Also, all the tools that successfully implement base editing in plant organelles are based on the dimeric TALE-linked deaminase architecture (Li et al. 2021; Nakazato et al. 2021, 2022, 2023; Mok et al. 2022). Monomeric TALE-linked adenine deaminase (AD; it is originally called mTALED, but we call it mTALEAD in this study to distinguish it easily from TALECD, the cytidine deaminase that catalyzes C-to-T) has only been reported to achieve A-to-G base editing in human mitochondrial DNA (Cho et al. 2022). mTALEAD consists of TALE arrays, a deoxyadenosine deaminase variant TadA8e engineered from *Escherichia coli* TadA that catalyzes A-to-G, and a catalytically inactive cytidine deaminase DddA_tox_ E1347A. The DddA_tox_ E1347A was set to open the double-stranded DNA for easy access and for the activity of TadA8e, which is known to act on single-stranded DNA rather than double-stranded DNA. Monomeric base editors are worth developing in plants because they are half the length of sequences recognized by the dimeric editors but are accurate enough to recognize the individual sites in the small organellar genomes of a few hundred kilobytes. Monomeric-based editors also do not require the simultaneous expression of 2 molecules with multiple or single-complicated dual expression vectors.

To test the feasibility of monomeric TALE-based base editors in plant organelles and to induce A-to-G conversion in plant mitochondrial genomes, we attempted to induce A-to-G conversion in NADH dehydrogenase subunit 7 (*nad7*) and adenosine triphosphatase (ATPase) subunit 6-2 (*atp6-2*) in mitochondria and 16S ribosomal RNA (*16S rRNA*) in the chloroplast of Arabidopsis (*Arabidopsis thaliana*) using mTALEADs.

## Results

### Generation of A**-**to**-**G conversion in *rrn 16S*, *nad7*, and *atp6-2*

**Figure 1.**
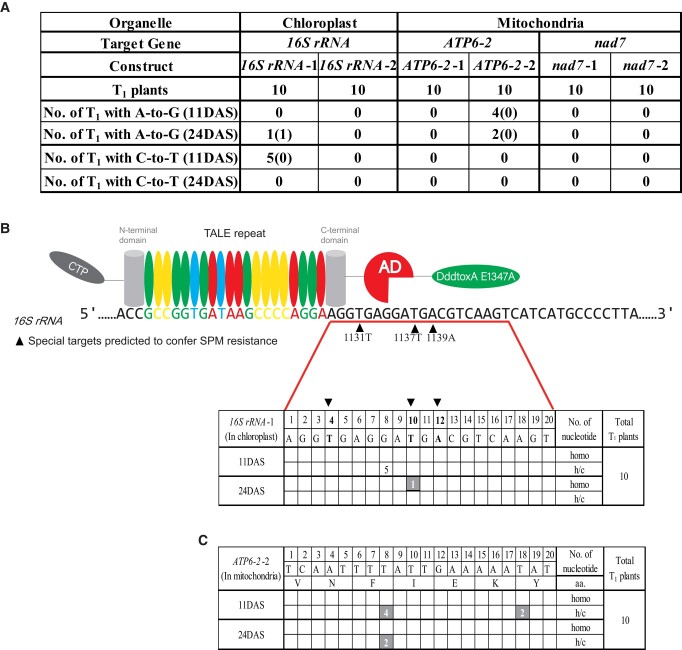
Genotyping of T_1_ plants. **A)** Numbers of T_1_ plants with A-to-G or C-to-T mutation for 6 constructs. The numbers in parentheses represent the number of T_1_ plants with homoplasmic substitution. **B)** Schematic of the mTALEAD construct of *16S rRNA*-1 and the number of T_1_ plants with bases edited and their positions for the construct *16S rRNA*-1 at 11 and 24 DAS. The triangles indicate the special base-editing targets predicted to confer spm^r^. CTP, chloroplast targeting peptide; TALE arrays: DNA-binding sequence; AD, adenine deaminase TadA8e; DddA_tox_ E1347A, a catalytically inactive cytidine deaminase; homo, homoplasmic substitution. The number of T_1_ plants with A-to-G conversion is marked in shadow, and the nonshadow number indicates C-to-T conversion. **C)** Base editing results in the target of 11 and 24 DAS T_1_ plants of the construct *atp6-2*-2.

**Figure 2.**
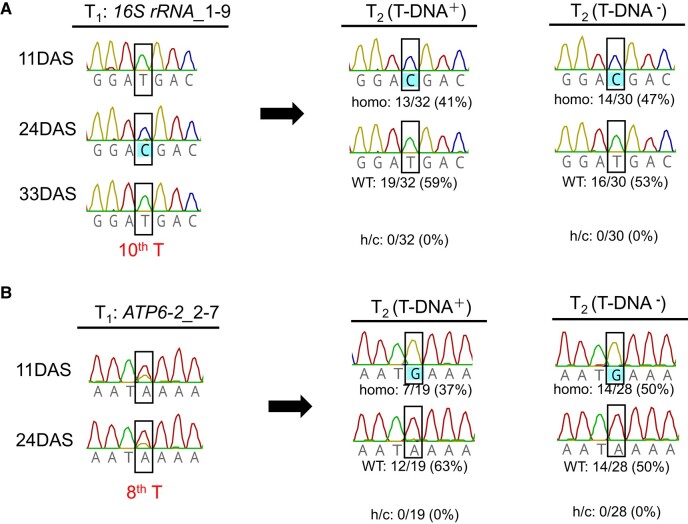
Genotyping of T_1_s and their progeny T_2_ plants for *16S rRNA*-1-9 and *atp6-2-2*-7. **A)** Number of T_2_ plants with wild-type (WT) or homoplasmic (homo) A-to-G conversion at the target 1137T, the 10th T of *16S rRNA-1* with or without T-DNA. **B)** Number of T_2_ plants with WT or homoplasmic A-to-G conversion at the target, the 8th T of *atp6-2*-2-7 with or without T-DNA. Target sites are bordered.

As targets for adenine base editing, we selected 3 sites (1131T, 1137T, and 1139A) in *16S rRNA* in the plastid genome, which are predicted to confer spectinomycin resistance (spm^r^) from the homologous sequences in the bacterial information (Miyazaki and Kitahara 2018; Supplementary Table S1). To simplify, both conversions of T-to-C and A-to-G are basically described as A-to-G in this study. In the mitochondrial genome, we selected genes *nad7* and *atp6-2*. An isoform of the ATP synthase subunit 6 gene, *atp6-2*, was chosen as a nonlethal target, without any genetic burden, because of its redundancy of genes in Arabidopsis ecotype Columbia (Arimura et al. 2020). Two vectors were constructed on either side of the target region (*nad7*-1/2, *atp6-2-*1/2, and *16S rRNA*-1/2) for each target gene (Supplementary Fig. S1). Ten T_1_ progenies were initially genotyped for each construct, and among the 6 constructs, only *16S rRNA*-1 and *atp6-2*-2 caused A-to-G conversion in the targeted region (Fig. 1A). For *rrn 16S rRNA*, no targeted A-to-G conversions were observed from leaves at 11 d after stratification (DAS), but the targeted base 1137T appeared to be homoplasmically (homo) substituted (10th T, which refers to the 10th T away from the TALE binding sequence) in a leaf sample of 1 plant with the vector *16S rRNA*-1 at 24 DAS. At the same time, five 11 DAS plants had heteroplasmic and/or chimerical (h/c; i.e. not homoplasmic) C-to-T conversion, but they were all not detected in any leaves at 24 DAS (Fig. 1, A and B), suggesting that C-to-T induced by the catalytically inactive cytidine deaminase DddtoxA E1347A (Cho et al. 2022) is not stable. The 3rd sequencing (33 DAS) was performed to see the genotype of this sole homo T_1_ mutant *16S rRNA*-1-9, and it showed the wild-type base (Fig. 2A), suggesting that the homoplasmic mutation occurred at some parts as a chimera in a plantlet. Compared with *16S rRNA*-1, 4 T_1_ plants with minor heteroplasmic or chimerical (h/c) A-to-G conversion at 8th T were found in *atp6-2*-2 at 11 DAS, and 2 of them (*atp6-2-*2-7 and *atp6-2*-2-9) also showed the A-to-G conversion in the leaves sampled at 24 DAS (Fig. 1A). Encouraged by this result, more T_1_ seeds were sowed and genotyped (Supplementary Fig. S2). This time A-to-G conversion was observed in 4 constructs: *16S rRNA*-1, *16S rRNA*-2, *atp6-2*-2, and *nad7*-1 (Supplementary Fig. S2A), and 13 of 46 T_1_ plants showed h/c A-to-G conversion at the 8th T in *atp6-2*-2 (Supplementary Fig. S2B). Eight plants with *nad7*-1 showed targeted A-to-G base editing at the 12th T and 17th T, but 7 of them except for *nad7*-1-22 had the wild-type base at these positions in another leaf sampled at 24 DAS (Supplementary Fig. S3). Collectively, 4 of 6 mTALEAD constructs functioned in plastids and mitochondria, and *atp6-2*-2 showed stable and the highest editing efficiency at the 8th T of the targeted sequence.

### Inheritance of mutations by seed progeny

To test whether the A-to-G mutations induced by mTALEADs can be inherited by seed progeny, we genotyped 62 T_2_ plants from a T_1_ line *16S rRNA*-1-9 and 47 T_2_ plants from a T_1_ line *atp6-2*-2-7, with and without transfer DNA (T-DNA) insertion in their nuclei. All of the h/c A-to-G conversions in T_1_ plants were segregated to the wild-type base or homo conversion in T_2_ progeny for both genes (Fig. 2), and in the case of T-DNA (i.e. no T-DNA), the homo conversion accounts for 47% for *16S rRNA* (Fig. 2A) and 50% for *atp6-2* (Fig. 2B). No h/c genotype was observed for both genes in the T_2_ generation. These results suggested that the A-to-G conversion in the plastid or mitochondrial genomes in T_1_ parents induced by mTALEADs can be inherited and fixed to homoplasmy, independently of the inheritance of the mTALEAD expression cassettes in the nuclei.

### Off-target analysis

**Figure 3.**
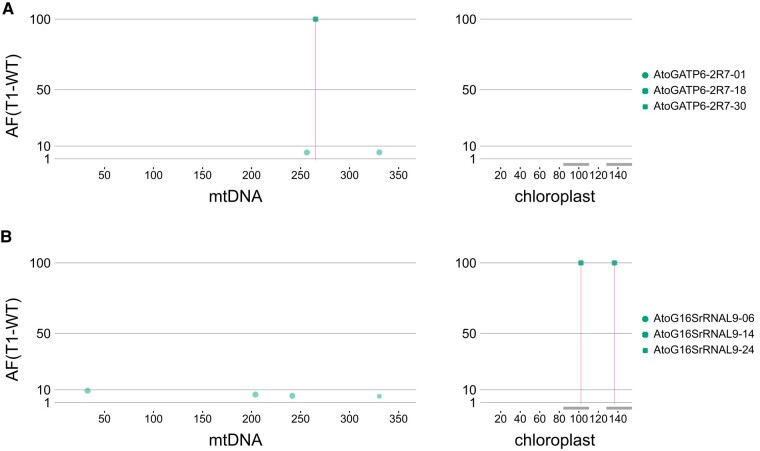
On-target and off-target SNPs in mitochondrial genomes and chloroplast genomes in 6 representative T_2_ plants (3 progenies of 2 T_1_ lines of *atp6-2*-2 **A)** and *16S rRNA*-1 **B).** None of these plants contained mTALEAD genes. The *x*- and *y*-axes show the positions and frequencies of the mutated SNPs that differed by at least 5% from the reference genomes BK010421.1 (mitochondria) and AP000423.1 (chloroplast). AF was calculated as AF_mu_−AF_WT_, where AF_mu_ is an AF of a SNP of each mutant and AF_WT_ is the average of the AFs of the same SNP of 3 WT plants.

Off-target effects of mTALEADs on the plastid and mitochondrial genomes were checked by using total DNA from 6 homoplasmically base-edited T_2_ null segregants (3 individual plants of each segregant for *atp6-2*-2-7 and *16S sRNA-*1-9) and 3 Col-0 via Illumina next-generation sequencing (NGS; Fig. 3; Supplementary Table S2). No dominant off-target point mutations were detected in all the T_2_ lines. mTALEAD targeting *16S rRNA*-1 introduced the targeted mutation with 100% allele frequency (AF) in the chloroplast genome of *A. thaliana*, and similar results were obtained for mTALEAD targeting *atp6-2* in the mitochondrial genome. In addition to the target sites, mTALEAD exhibited minor frequent (<10% of the reads) off-target A-to-G mutations/polymorphisms at 5 sites in the mitochondrial genomes. However, they were also detected in the wild-type plants with a similar frequency. Four of the 5 off-target mutations/polymorphisms were likely to be artifacts caused by the nuclear-encoded mitochondrial DNA sequences in chromosome 2 (Naish et al. 2021; Fields et al. 2022; Wang et al. 2022; Supplementary Table S2). All the results indicated that mTALEADs could specifically convert T:A to C:G in the target windows without substantial off-targets in organellar genomes.

### spm^r^ test of T2 progenies of *16S rRNA*-1

Verification of spm resistance in T_2_ progenies of *16S rRNA*-1 with a homoplasmic substitution of T1137C was performed. T_2_ seeds of *16S rRNA*-1-9 were sowed on plates containing 50 mg L^−1^ of spm. The same number of Col-0 seeds and T_3_ seeds of *16S rRNA* C1015T spm^r^ mutants (Nakazato et al. 2021) were sowed. Consequently, all the T_2_ progenies of *16S rRNA*-1-9 showed a spm-sensitive (spm^s^)–like phenotype like wild-type plants (Supplementary Fig. S4A), even in the lower concentration of spm, 5 and 0.5 mg L^−1^ (Supplementary Fig. S4, B and C). T1137C in *16S rRNA* may confer no spm^r^ or very weak spm^r^ in *A. thaliana*, which is also consistent with the previous study that reported that the corresponding 1137T site in *E. coli 16S rRNA* U1189 conferred a modest spm^r^ to *E. coli* (Miyazaki and Kitahara 2018).

### Shifting the TALE arrays to obtain A**-**to**-**G conversions at 1131T and 1139A in *16S rRNA*

**Figure 4.**
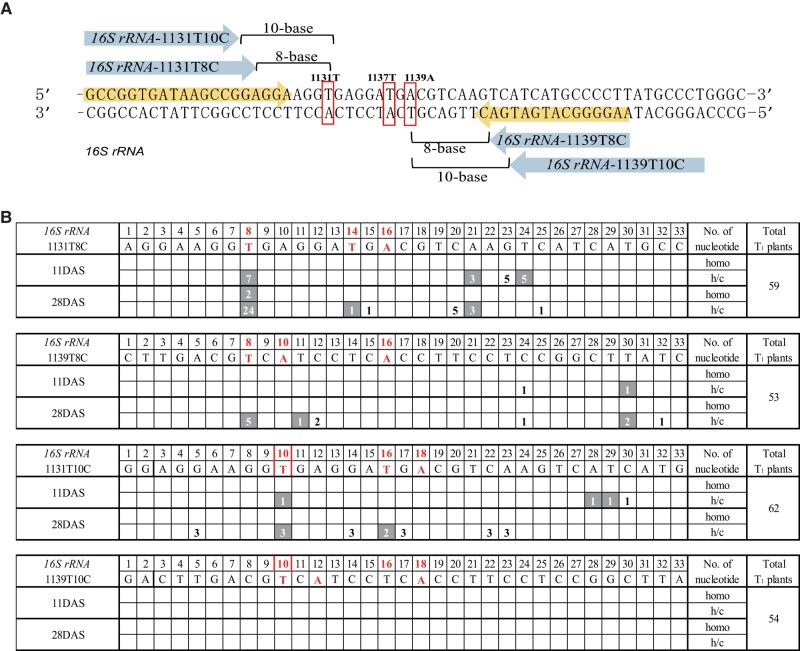
Genotyping of T_1_ plants of *16S rRNA*_1131T and *16S rRNA*_1139A. **A)** Shifting of TALE arrays to make the new target bases (1131T and 1139A) at the 8th or 10th T. TALE arrays of 4 new constructs are showed and the 1st tested constructs (*16S rRNA*-1 and *16S rRNA*-2) are marked within the sequence. **B)** Base editing results in the target windows of *16S rRNA* of 11 or 28 DAS T_1_ plants for 3 new constructs. The number of T_1_ plants with A-to-G conversion is marked in shadow, and the nonshadow number indicates C-to-T conversion. Vacant means 0. homo, homoplasmic substitution.

Since the substitution of *16S rRNA*_1137T conferred very weak spm^r^ in *A. thaliana*, we sought to induce A-to-G substitution into 2 other sites in *rrn 16S rRNA* that are expected to confer stronger spm resistance in *A. thaliana*: 1131T and 1139A (O’Neill et al. 1993; Criswell et al. 2006; Filipenko et al. 2011; Miyazaki and Kitahara 2018). Although we succeeded in mutating the 10th T in the 1st trial in *rrn 16S rRNA*, it is suggested from the previous data (Fig. 1B; Supplementary Fig. S2B) that the 8th T is the most efficient site for the introduction of A-to-G conversion by mTALEADs. Therefore, to induce A-to-G conversion in 1131T and 1139A efficiently, 4 new mTALEADs were designed by placing 1131T and 1139A at the 8th or 10th positions of T. They are as follows: (i) *rrn 16S rRNA*_1131T8C, (ii) *rrn 16S rRNA*_1131T10C, (iii) *rrn 16S rRNA*_1139T8C, and (iv) *rrn 16S rRNA*_1139T10C (Fig. 4A). T_1_ plants with each of the new constructs were sowed, and their genotype was checked twice (11 and 28 DAS). As shown in Fig. 4B, plenty of A-to-G conversions were found at the 8th T in *16S rRNA*_1131T8C. In *16S rRNA*_1139T8C, 5 h/c A-to-G conversions at the 8th T were detected out of 53 T_1_ plants. We also observed 3 h/c A-to-G conversions at the 10th T at 28 DAS in *16S rRNA*_1131T10C. There were no A-to-G conversions detected in *16S rRNA*_1139T10C in all the tested 54 T_1_ plants (Fig. 4B) In a word, mTALEADs exhibited higher editing efficiency at the 8th T than at the 10th T, and we successfully obtained mutants at 1131T and 1139A of *16S rRNA* via the strategy of shifting TALE arrays to place 1131T and 1139A at the 8th T.

### spm^r^ test of *16S rRNA*_T1131C and *16S rRNA*_A1139G

**Figure 5.**
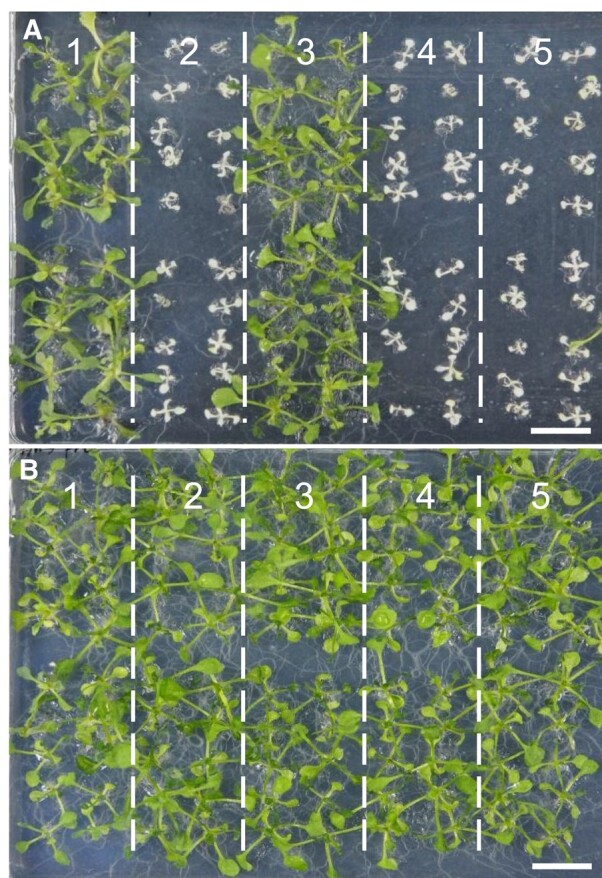
Seventeen DAS phenotypes of 3 *16S rRNA* mutants. **A)** The 1/2 MS medium containing 10 mg L^−1^ spectinomycin. **B)** The 1/2 MS medium. 1: T_3_ plants of *16S rRNA-*1139T8C, line 30-4. 2: T_2_ plants of *16S rRNA-*1131T8C, line 22. 3: Positive control T_3_ seeds of *16S rRNA*_C1015T. 4: Wild-type Col-0 plants. 5: T_2_ plants of *16S rRNA-*1137-1, line 9. Scale bar, 1 cm.

To check whether spm^r^ was conferred by substitution of 1131T and 1139A of *16S rRNA*, 20 seeds were sowed for each *16S rRNA* mutant (*16S rRNA*_T1131A, *16S rRNA*_A1139G, and *16S rRNA*_T1137A) on 1/2 MS medium with or without 10 mg L^−1^ spm (Fig. 5, A and B). Consequently, all the T_2_ seeds of *16S rRNA*_1131T8C-22 (Fig. 5, Line 2) showed an obvious spm-sensitive phenotype like *16S rRNA*_1137T8C and the wild-type Col-0 plants. On the other hand, all of the tested 20 plants with *16S rRNA*_A1139G (Fig. 5, Line 1) exhibited spm^r^, which is consistent with the previous study that induced A-to-G conversion at 1139A by dimeric TALEADs (Mok et al. 2022). The spm^r^ test indicated that the substitution at 1139A in the *16S rRNA* was capable of conferring spm^r^ in *A. thaliana*, while the substitutions at 1131T and 1137T, which provided 8-fold and 32-fold resistance to *E. coli*, respectively, conferred little resistance to Arabidopsis (Supplementary Table S1).

### The most efficient base site for inducing A-to-G conversion via mTALEADs

**Figure 6.**
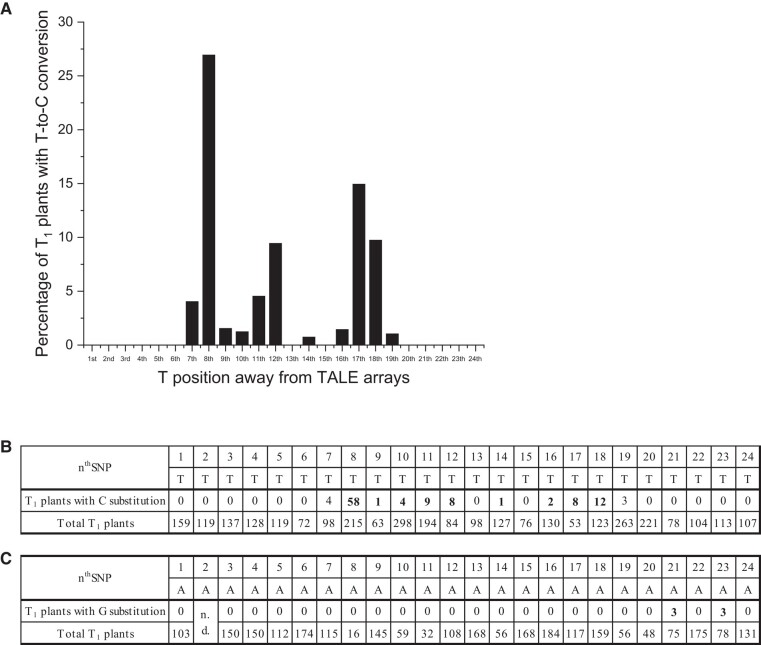
The percentage of T_1_ plants with C or G substitution in the different positions of T or A induced by mTALEADs in this study. **A)** The percentage of T_1_ plants with substitution in the *n*th T, summarized from **B). B)** The number of total detected T_1_ plants and T_1_ plants with C substitution in different T positions, accumulated from all the results from all constructs examined. **C)** The number of total detected T_1_ plants and T_1_ plants with G substitution in different A positions.

Last but not least, we roughly reassessed editing efficiency at different T (*n*th T, where *n* refers to the number of bases in the targeting window away from TALE arrays) and A (*n*th A) positions in the sense strand, based on the results of base editing in T_1_ plants from the constructs of mTALEADs (Supplementary Table S3; Fig. 6). Constructs that did not bring any mutations (even C-to-T) were excluded from the counting. It is notable that in 215 T_1_ plants that had T at the 8th base, 58 had homo or h/c A-to-G conversion in the opposite strand, which is the highest ratio (58/215, 27%) among all the T/A sites (Fig. 6, A and B). Fifteen percent of the T_1_ plants that had T at the 17th base had A-to-G conversion at the 17th T. It is also unexpected that mTALEADs displayed very little editing effects for As in the sense strand. In 75 and 78 T_1_ plants that had As at the 21st and 23rd bases, respectively, only 3 plants had A-to-G mutation at these As (Fig. 6C). This result suggests that placing the targeted base at the 8th T from the TALE arrays would maximize the editing efficiency of the base in plant organellar DNA.

## Discussion

In this study, we succeeded in targeted A-to-G editing in Arabidopsis in both mitochondria and chloroplasts by monomeric TALEADs under the control of the RPS5A promoter via Agrobacterium-mediated transformation. Analyses of T_2_ progenies of *16S rRNA* and *atp6-2* indicated that the induced A-to-G conversion could be stably inherited independently of the inheritance of the mTALEAD expression vector in nuclei.

For sequences without T at the 8th base, mTALEADs showed little editing efficiency (Fig. 1B; Supplementary Fig. S2), whereas for sequences with T at the 8th base, 27% of the tested T_1_ plants had A-to-G conversions at the 8th T (Fig. 6). However, there is also the case in which mTALEAD showed low editing frequency at the 8th T (e.g. *16S rRNA*-1139T8C, Fig. 4), which might be caused by the differences of surrounding sequences and/or TALE repeats/protein structure of the enzymes. Such differences should be characterized more in the future, which will contribute to developing more accurate and useful A-to-G base editors.

Concurrent C-to-T and A-to-G edits were observed in *16S rRNA* and *atp6-2* in this study (Supplementary Fig. S5). In addition, while UGI-free dimeric TALEDs induced A-to-G edits without C-to-T edits in human mitochondria (Cho et al. 2022), they induced C-to-T edits in addition to A-to-G edits in protoplasts and all examined plants (Mok et al. 2022). It is speculated that uracil bases (the intermediates during C-to-T conversion) produced by UGI-free TALEDs in plant organelles are less efficiently removed by endogenous uracil DNA glycosylase than in human mitochondria, as proposed by a previous study (Kim and Chen 2023). The C-to-T edits observed in this study were likely to be caused by the full-length catalytically inactive cytidine deaminase DddA_tox_ E1347A, which was engineered to act and open double-stranded DNA, making it more accessible to the AD TadA8e that operates on single-stranded DNA. Using DddA_tox_ with multiple point mutations at active sites may help suppress C-to-T editing. It is also a future task to develop ABEs that induce only A-to-G in plant organelles.

Many T_2_ plants inherited homoplasmic A-to-G mutations from their heteroplasmically or chimerically edited parental T_1_ mutants (Fig. 2B). The obtained null segregant T_2_ plants will be good materials to reveal the effect of targeted A-to-G base editing on phenotypes. In addition, these null segregant T_2_ plants are suitable for practical use because they are exempt from Genetically Modified Organism regulations in some countries. The rapid segregation of homoplasmic base–edited T_2_ plants in this study would compensate for, in some cases, the low efficiency of A-to-G observed in the T_1_ generation. Such rapid segregation of homoplasmy in T_2_ plants is supported by the study showing that heteroplasmic sorting out occurs relatively rapidly, even in 1 to 2 generations for both mitochondrial and plastid genomes (Broz et al. 2022).

Taken together, our study demonstrated the feasibility of mTALEADs in plant organelles. The inclined high efficiency of base editing at the 8th T (Fig. 6) contributes to the design of targeted editing enzymes with high accuracy and suppression of unintentional off-target mutations. This work contributes to the future development and application of monomeric organellar genome editing tools in plant organelles.

## Materials and methods

### Plant materials

Arabidopsis (*A. thaliana*) Columbia-0 (Col-0) and transgenic plants were grown at 22 °C and under long-day conditions (16 h light, 8 h dark). Col-0 seeds were sown on 1/2 MS medium (pH 5.7) containing 2.3 g L^−1^ of MS Plant Salt Mixture (Wako), 500 mg L^−1^ of MES, 10 g L^−1^ of sucrose, 1 mL L^−1^ of Plant Preservative Mixture (Plant Cell Technology), 1 mL L^−1^ of Gamborg's Vitamin Solution (Sigma-Aldrich) and 5 g L^−1^ of agar. Seedlings at 2 to 3 wk old were transferred to Jiffy-7 (Jiffy Products International) in the greenhouse at 22 °C under long-day conditions and thereafter subjected to Agrobacterium transfection.

### Designing the TALE binding sequence and plasmid construction

The TALE targeting sequence was designed to be on one side of the AD targeting window, and we prepared 2 TALEAD vectors for each target gene (Supplementary Fig. S1). The length of TALE-binding sequences was designed between 15 and 20 bp (Supplementary Table S3), so that TALE repeats would specifically recognize and bind to the targeting sequence. ptpTALEADs or mtTALEADs in Ti-plasmids for each target was constructed by using a Platinum Gate assembling kit (Sakuma and Yamamoto 2016) and multisite Gateway (Thermo Fisher) as described in our previous study of mitochondria-targeted TALEN (Kazama et al. 2019). The DNA-binding domains of ptpTALEAD or mtTALEAD were assembled with the Platinum Gate TALEN system based on our previous study (Nakazato et al. 2021). The construct was composed of DNA-recognizing sequence TALE arrays, a deoxyadenosine deaminase variant for catalyzing A-to-G conversion, termed TadA8e, engineered from the *E. coli* TadA (Richter et al. 2020; shown as AD in the figures) and a catalytically inactive, nontoxic, full-length cytidine deaminase DddA_tox_ variant with an active-site E1347A mutation (Fig. 1A). The N terminus of the mTALEADs was linked to a chloroplast targeting signal peptide of the *A. thaliana* RecA1 protein (51 aa; Cerutti et al. 1992; Cao et al. 1997) or the mitochondrial targeting peptide of the Arabidopsis ATPase delta prime subunit (Arimura et al. 2020) to introduce the expressed mTALEAD proteins into chloroplast or mitochondria. Each CD half and UGI coding sequence in the previous vectors of ptpTALECDs used for assembly-step2 was replaced in advance by TadA8e and DddA_tox_ E1078K coding sequences. TadA8e and DddA_tox_ E1078K coding sequences were designed to encode the same amino acid as Cho's experiment (Cho et al. 2022) and artificially synthesized by Twist Bioscience, with restriction enzyme cutting sites bglII and pstI at the beginning and end of the sequence. Both TadA8e coding sequence and E1 vector pENTR_L1-L4_HD_G1397-DddtoxA-N were subjected to double enzyme digestion with bglII and pstI and ligated by Quick Ligase Enzyme. The reading frame in the assembled entry vectors including the TALE sequence and TadA8e and DddA_tox_ E1078K coding sequences were transferred into the Ti plasmid (Nakazato et al. 2021) by transferring DNA fragments between different plasmids or vectors via specific recombination sites attL sites and attR sites (LR) reaction with LR Clonase II Plus enzyme (Thermo Fisher Scientific). All primers used for sequencing to verify that the vectors in this study were constructed correctly are listed in Supplementary Table S4. All plasmids have been deposited in Addgene (https://www.addgene.org/), and all the vectors are available in Addgene (ID 210829 to 210838). The complete sequences for all plasmids are also available in GenBank (https://www.ncbi.nlm.nih.gov/) under accession numbers OR842973 to OR842982.

### Plant transformation and screening transformants

Col-0 plants were transformed using the floral dip method (Clough and Bent 1998) with the *Agrobacterium tumefaciens* strain C58C1 that harbored one of the transformation vectors described above. The obtained T_1_ seeds were selected by their seed-specific fluorescence of GFP (Shimada et al. 2010). GFP-positive seeds were sown on the 1/2 MS medium further containing 125 mg L^−1^ of claforan. During the spm^r^ test, T_2_ seeds of *16S rRNA* were sowed on the 1/2 MS medium containing 0.5/5/10/50 mg L^−1^ of spectinomycin, respectively, and other T_2_ seeds were sowed on the 1/2 MS medium.

### Genotyping T_1_ and T_2_ plants

For Sanger sequencing, PCR was conducted using KOD One PCR Master Mix (TOYOBO) with roughly extracted DNA from an emerging leaf or cotyledon at 7/11 DAS and uppermost rosette leaf at 24/28/33 DAS with standard protocols. Roughly extracted DNA was performed by placing one leaf in 50 *µ*L of the Plant Very Rapid PCR Isolation Buffer (containing 5 mmol g^−1^ EDTA with pH = 8.0 and 0.1 mol g^−1^ Tris HCl with pH = 9.5) after a 15-min treatment at 98 °C. DNA sequences adjacent to the target DNA were amplified with primer sets (Supplementary Table S4). Purified PCR products were subjected to Sanger sequencing (Eurofins Genomics) to detect substitution of the targeted bases. The data were analyzed with Geneious prime (v.2022.1.1). Total DNA was extracted from mature leaves of Arabidopsis with the DNeasy Plant Pro Kit (QIAGEN), and we used the same analyzing method for Illumina NGS analyses with the reported study (Nakazato et al. 2021). For each individual plant, total DNA was sequenced using the Illumina NovaSeq 6000 platform. As a preprocess for analysis, low-quality and adaptor sequences in the reads were trimmed using Platanus_trim v.1.0.7 (http://platanus.bio.titech.ac.jp/pltanus_trim). Pair-end reads of each strain were mapped to reference sequences (AP000423.1 and BK010421.1) using BWA (v.0.7.12)37 in a single-ended mode. We filtered out inadequate mapped reads with mapping identities ≤97% or alignment cover rates ≤80%. Single nucleotide polymorphisms (SNP s) were then called using samtools mpileup command (-uf -d 30000 -L 2000) and bcftools call command (-m -A -P 0.1)38. We finally listed positions in which variants with AFs ≥ 0.1 were detected in at least one strain (Fig. 3). SNP calls with AFs ≥ 0.01 were also performed for positions with read depths ≥500 (Supplementary Table S2).

### Image processing

Plant images were taken with a digital camera (OLYMPUS OM-D E-M5) and processed with Adobe Illustrator 2020.

### Accession numbers

Sequence data from this article can be found in the GenBank/EMBL data libraries under accession numbers OR842973 to OR842982.

## Supplementary Material

kiad678_Supplementary_Data

## Data Availability

The data underlying this article are available in Addgene (https://www.addgene.org/) and all the vectors are available in Addgene (ID 210829–210838). The complete sequences for all plasmids are also available in Genbank (https://www.ncbi.nlm.nih.gov/), with accession numbers OR842973 –OR842982.

## Supplementary data

The following materials are available in the online version of this article.

**Supplementary Figure S1.** Three targeting sequences and 6 mTALEADs constructs.

**Supplementary Figure S2.** Second genotyping of T_1_ plants.

**Supplementary Figure S3.** Sanger sequence of 8 T_1_ plants with an A-to-G conversion in *nad7*-1.

**Supplementary Figure S4.** T_2_ seeds of *16S rRNA*_T1137C on spm plates.

**Supplementary Figure S5.** The percentage of T_1_ plants with T or A substitution in the different positions of C or G induced by mTALEADs in this study.

**Supplementary Table S1.** A summary of spm resistance–related sites.

**Supplementary Table S2.** Analyses of on-Target and off-Target results of mTALEADs.

**Supplementary Table S3.** Constructs of mTALEADs.

**Supplementary Table S4.** Primers used in this study.

## Dive Curated Terms

The following phenotypic, genotypic, and functional terms are of significance to the work described in this paper:

ATP CHEBI: CHEBI:15422

CTP CHEBI: CHEBI:17677

NADH CHEBI: CHEBI:16908
