# Supplementary material for: Targeted A-to-G base editing in the organellar genomes of Arabidopsis with monomeric programmable deaminases
Source: Plant Physiol. 2023 Dec 21;194(4):2278–87. doi: 10.1093/plphys/kiad678 (PMC10980515; doi:10.1093/plphys/kiad678)
Supplement: kiad678_Supplementary_Data [file kiad678_supplementary_data.pdf]

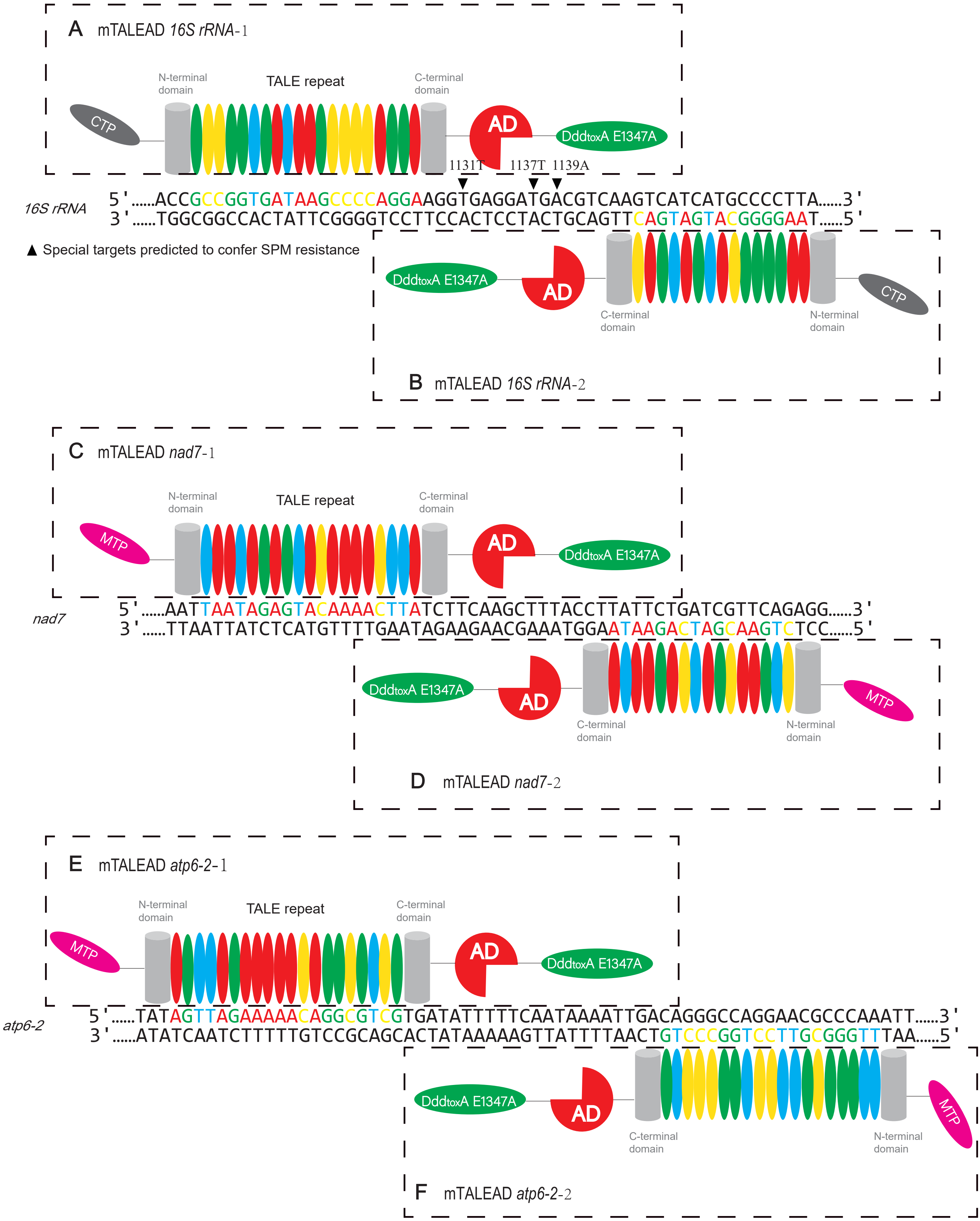

Supplemental Fig. S1 Three targeting sequences and 6 mTALEADs constructs. (A-B) Two mTALEADs of 16*S rRNA*. CTP:chloroplast targeting peptide. TALE arrays: DNA binding sequence. AD: adenine deaminase TadA 8e. $DddA_{tox}$ E1347A: a catalytically inactive cytidine deaminase for making double-stranded DNA more accessible to TadA 8e, which originally acts on single-stranded RNA. MTP: mitochondria targeting sequence. **(C-D)** Two mTALEADs of *nad7*. **(E-F)** Two mTALEADs of *atp6-2*.

A

Organelle	Chloroplast		Mitochondria			
Target Gene	*16S rRNA*		*ATP6-2*		*nad7*	
Construct	*16S rRNA*-1	*16S rRNA*-2	*ATP6-2*-1	*ATP6-2*-2	*nad7*-1	*nad7*-2
T_1 plants	34	25	18	46	43	43
No. of T_1 with A-to-G (7DAS)	1	2	0	19	8	0
No. of T_1 with C-to-T (7DAS)	13	0	0	7	0	0

B

16S rRNA-1 (In chloroplast)	1	2	3	4	5	6	7	8	9	10	11	12	13	14	15	16	17	18	19	20	21	22	23	24	25	26	27	28	29	30	31	32	33	34	No. of nucleotide	Total T_1 plants
	A	G	G	T	G	A	G	G	A	T	G	A	C	G	T	C	A	A	G	T	C	A	T	C	A	T	G	C	C	C	C	T	T	A		
7DAS																																			homo	34
					1			13																									1		h/c	

16S rRNA-2 (In chloroplast)	1	2	3	4	5	6	7	8	9	10	11	12	13	14	15	16	17	18	19	20	21	22	23	24	25	26	27	28	29	30	31	32	33	34	No. of nucleotide	Total T_1 plants
	T	T	G	A	C	G	T	C	A	T	C	C	T	C	A	C	C	T	T	C	C	T	C	C	G	G	C	T	T	A	T	C	A	C		
7DAS																																			homo	25
																													2						h/c	

ATP6-2-2 (In mitochondria)	1	2	3	4	5	6	7	8	9	10	11	12	13	14	15	16	17	18	19	20	21	22	23	24	25	26	27	28	29	30	31	32	33	34	No. of nucleotide	Total T_1 plants
	T	C	A	A	T	T	T	T	A	T	T	G	A	A	A	A	A	T	A	T	C	A	C	G	A	C	G	C	C	T	G	T	T	T		46
	V			N			F			I			E			K			Y			H			D			A			C		F		aa.	
7DAS																																			homo	
								13			3							13			7														h/c	

nad7-1 (In mitochondria)	1	2	3	4	5	6	7	8	9	10	11	12	13	14	15	16	17	18	19	20	21	22	23	24	25	26	27	28	29	30	31	32	33	34	No. of nucleotide	Total T_1 plants
	T	C	T	T	C	A	A	G	C	T	T	T	A	C	C	T	T	A	T	T	C	T	G	A	T	C	G	T	T	C	A	G	A	G		43
	Y		L			Q			A			L			P			Y			S			D			R			S			D		aa.	
7DAS																																			homo	
												8					8																		h/c	

Supplemental Fig. S2 2nd genotyping of T_1 plants. (A) 2nd sowing of T_1 plants with A-to-G or C-to-T mutation for 6 constructs. **(B)** Base editing result in the target windows of 7 DAS T_1 plants for 4 constructs. DAS: days after stratification. homo: homoplasmic substitution. h/c: heteroplasmic and/or chimerical substitution. The number of T_1 plants with A-to-G conversion are marked in shadow. Non-shadow number indicates C-to-T conversion.

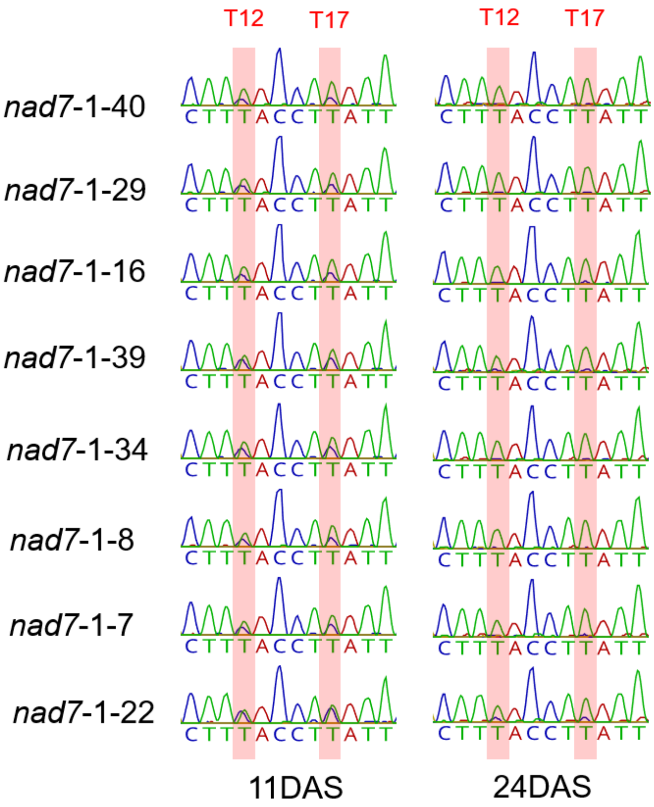

Supplemental Fig. S3 Sanger sequence of 8 T_1 plants with an A-to-G conversion in *nad7*-1.

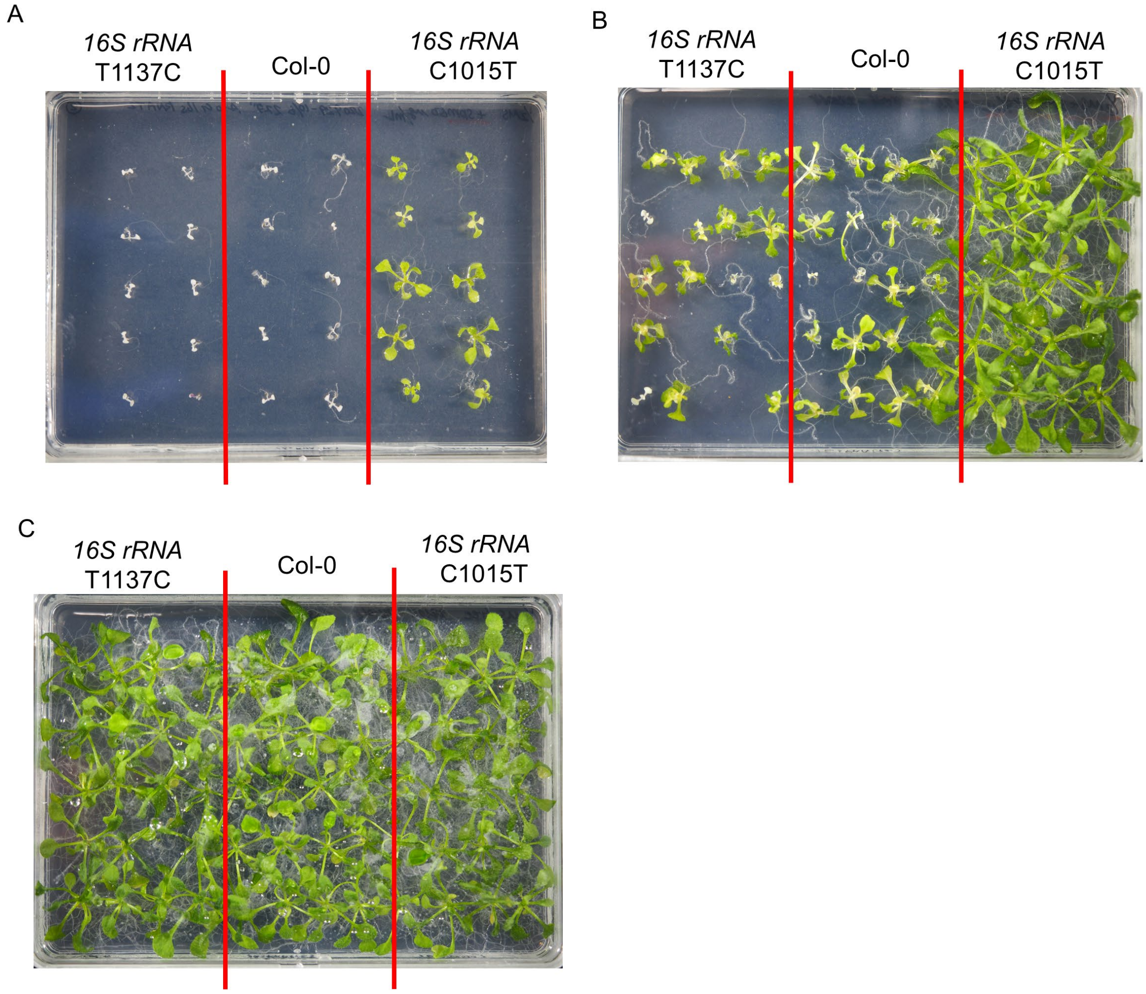

Supplemental Fig. S4 T_2 seeds of *16S rRNA*_T1137C on spm plates. (A) 50 mg l^{-1} spm plate. **(B)** 5 mg l^{-1} spm plate. **(C)** 0.5 mg l^{-1} spm plate. Seeds of Col-0 were sowed as negative control and seeds of *16S rRNA*_C1015T were sowed as positive control.

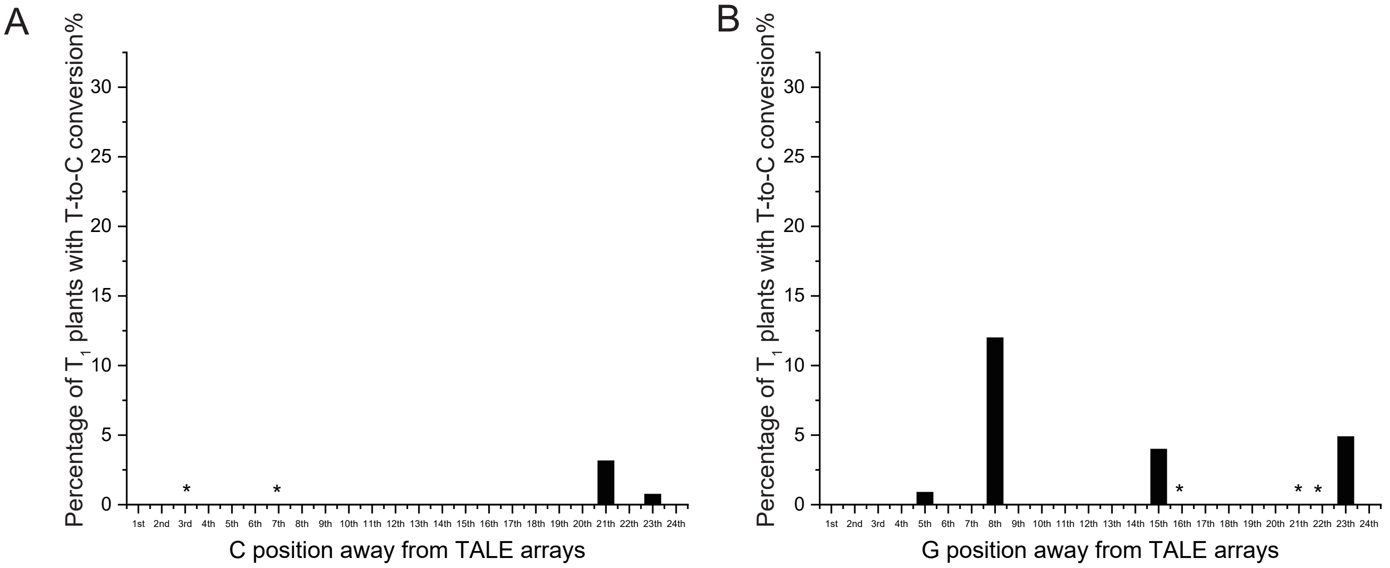

C

n^{th}SNP	1	2	3	4	5	6	7	8	9	10	11	12	13	14	15	16	17	18	19	20	21	22	23	24
	C	C	C	C	C	C	C	C	C	C	C	C	C	C	C	C	C	C	C	C	C	C	C	C
T_1 plants with T substitution	0	0	n. d.	0	0	0	n. d.	0	0	0	0	0	0	0	0	0	0	0	0	0	0	0	0	0
Total T_1 plants	85	99		32	68	85		25	96	15	25	78	97	100	96	101	152	53	62	84	221	140	81	132

D

n^{th}SNP	1	2	3	4	5	6	7	8	9	10	11	12	13	14	15	16	17	18	19	20	21	22	23	24
	G	G	G	G	G	G	G	G	G	G	G	G	G	G	G	G	G	G	G	G	G	G	G	G
T_1 plants with A substitution	0	0	0	0	0	0	0	0	0	0	0	0	0	0	0	n. d.	0	0	0	0	n. d.	n. d.	0	0
Total T_1 plants	78	197	128	115	106	84	156	149	121	165	165	147	62	122	75		94	90	44	62			102	10

Supplemental Fig. S5 The percentage of T_1 plants with T or A substitution in the different positions of C or G induced by mTALEADs in this study. A. Percentage of T_1 plants with C-to-T substitution in n^{th} C, summarized from table C. **B.** Percentage of T_1 plants with C-to-T substitution in n^{th} G, summarized from table D. **C.** Number of total detected T_1 plants and T_1 plants with C-to-T substitution in different position's C, accumulated from the all the results from all constructs functioned. **D.** Number of total detected T_1 plants and T_1 plants with C-to-T substitution in different position's G. SNP: Single-Nucleotide Polymorphisms. *: no data (n. d.).

Supplemental Table S1 Summary of spm resistance-related sites.

Host / Bacteria	Position	Resistance	Host / Plants	Position	Resistance
E. coli MY205 (Miyazaki & Kitahara, 2018)	C1066U	>1024	*Arabidopsis. thaliana* (Nakazato I, *et al*. 2021)	C1015T	100 mg l^{-1}
E. coli MY205 (Miyazaki & Kitahara, 2018)	U1183C	1024	*Arabidopsis. thaliana* (this work)	T1131C	WT-like
E. coli MY205 (Miyazaki & Kitahara, 2018)	U1189C	256	*Arabidopsis. thaliana* (this work)	T1137C	WT-like
Borrelia burgdorferi (Criswell *et al*., 2006)	A1185G	>2200	*Arabidopsis. thaliana* (Mok Y G. *et al*. 2022)(this work)	A1139G	10 mg l^{-1}
			Daucus carota (Fillipenko *et al*., 2011)	A1139G	N/A
			Nicotiana plumbaginifolia (O'Neill *et al*., 1993)	A1139G	N/A

Supplemental Table S2 Analyses of on- and off-Target result of mTALEADs.

Genome	*atp6-2*	*16S rRNA*	POS	REF	ALT	SNP in NUMT	Allele frenquency								
							Col-0			*atp6-2*-2-7			*16S rRNA*-1-9		
							#1	#2	#3	#1	#2	#3	#1	#2	#3
chr		●	102148	T	C	-	0.0	0.0	0.0	0.0	0.0	0.0	**100.0**	**100.0**	**100.0**
		●	136501	A	G	-	0.0	0.0	0.0	0.0	0.0	0.0	**100.0**	**100.0**	**100.0**
mito			32738	T	C	C	8.1	5.6	9.0	6.4	9.0	3.1	25.6	9.4	9.1
			203930	A	G	G	5.8	6.7	6.9	5.8	8.5	8.0	20.5	8.4	6.9
			241619	A	G	G	7.7	8.3	10.5	8.3	7.2	8.3	24.7	11.4	9.6
			256423	T	C	C	5.7	7.2	6.3	13.5	7.3	6.9	17.7	10.2	13.6
	●		265283	A	G	A	0.0	0.0	0.0	**100.0**	**100.0**	**100.0**	0.0	0.0	0.0
			330351	A	G	A	4.6	5.2	7.0	12.6	7.0	6.6	14.9	8.6	14.1

● Targeted base that was induced with A-to-G

Individual plants showed 100% T:A pairs in all reads on the induced site and was marked in gray.

A-to-G refers to T:A pairs to C:G pairs, including cases that T or A on the coding strand

POS: Nucleotide position among the chloroplast (chl) genome or mitochondrial (mito) genome

REF: reference genome of wild type

ALT: Alternative base

SNP: Single-Nucleotide Polymorphism

NUMT: nuclear-encoded mitochondrial DNA sequences

Allele frequency refers to the percentage of an alternative base in all reads.

Supplemental Table S3 Constructs of mTALEADs.

Targeted gene	mTALEAD	TALE arrays (5' to 3')	A-to-G in T_1 transformants
16S rRNA	*16S rRNA*-1	GCCGGTGATAAGCCGGAGGA	✓
	16S rRNA-2	AAGGGGCATGATGAC	✓
nad7	*nad7*-1	CTGAACGATCAGAATA	✓
	nad7-2	TAATAGAGTACAAAACTTA	×
atp6-2	*atp6-2*-1	AGTTAGAAAAACAGGCGTCG	×
	atp6-2-2	TTGGGCGTTCCTGGCCCTG	✓
16S rRNA	*16S rRNA*_1131T8C	GCCGGTGATAAGCCGG	✓
	*16S rRNA*_1131T10C	GCCGGTGATAAGCC	✓
	*16S rRNA*_1139T8C	AAGGGGCATGATGA	✓
	*16S rRNA*_1139T10C	AGGGCATAAGGGGCATGAT	×

Supplemental Table S4 Primers used in this study.

Primer	Sequence (5'->3')	length	note	GC%	Tm	Product Length
*nad7*_Fw	TTTGCGAATGAATGCTGGGC	20	for amplifying target DNA fragments and genotyping	50	60.11	493
*nad7*_Rv	TTCCGGTGCACTCGCTTTAT	20		50	60.04	
*16sRNA*_Fw	GGAGCGGTGAAATGCGTAGA	20		55	60.46	762
*16sRNA*_Rv	AAGGTAACGACTTCGGGCAT	20		50	59.39	
*atp6-2WY*_Fw	CATTCCCGGAAAGACCACCT	20		55	59.67	410
*atp6-2WY*_Rv	CGGGAGCAAACTGGACCTTA	20		55	59.68	
pCR8-F1	TTGATGCCTGGCAGTTCCCT	20	for checking assembly-step 1 vectors	–		
TAL-F2	GGAGGCAGTGCATGCATGGC	20	for checking assembly-step 2 vectors			
TAL-R2	GGCGACGAGGTGGTCGTTGG	20				
pK7WG2_pOLE1_fsRv	CTAAGTAGGGTGCCGGGGAT	20	for checking assembly-step3 (final) vectors			
TALR1r	CCAACGACCACCTCGTCGCC	20				
pRPS5A_fsFw2	CCAGGGAACCTGTTAAACCG	20				
TALR2	GGCGACGAGGTGGTCGTTGG	20				
